# Evaluation and prioritisation of actions on food environments to address the double burden of malnutrition in Senegal: perspectives from a national expert panel

**DOI:** 10.1017/S1368980022000702

**Published:** 2022-08

**Authors:** Julien Soliba Manga, Adama Diouf, Stefanie Vandevijvere, Maty Diagne, Komlan Kwadjode, Nicole Dossou, El Hadji Momar Thiam, Ndeye Fatou Ndiaye, Jean-Claude Moubarac

**Affiliations:** 1 Division de l’Alimentation et de la Nutrition, Direction de la Santé de la Mère et de l’Enfant (DSME) du Ministère de la Santé et de l’Action Sociale, Rue FN 20, Dakar, Sénégal; 2 Département de Nutrition, TRANSNUT (Centre collaborateur OMS) et CRESP (Centre de Recherche en Santé Publique), Université de Montréal, 2405 Chemin de la Côte-Sainte-Catherine, Montréal H3T 1A8, Canada; 3 Laboratoire de Recherche en Nutrition et Alimentation Humaine (LARNAH), Département de Biologie Animale, Faculté des Sciences et Techniques, Université Cheikh Anta Diop de Dakar, Dakar, Sénégal; 4 Sciensano, Service of Lifestyle and Chronic Diseases, Brussels, Belgium; 5 Organisation des Nations Unies pour l’Alimentation et l’Agriculture, Bureau, Dakar, Sénégal; 6 Cellule de Lutte contre la Malnutrition, Primature, Dakar, Sénégal; 7 Institut Technologie Alimentaire, Dakar, Sénégal

**Keywords:** Public policy, Government actions, Policy index, Evaluation, Healthy food environments

## Abstract

**Objective:**

To evaluate the extent of implementation of public policies aimed at creating healthy eating environments in Senegal compared to international best practice and identity priority actions to address the double burden of malnutrition.

**Design:**

The Healthy Food Environment Policy Index (Food-EPI) was used by a local expert panel to assess the level of implementation of forty-three good practice policy and infrastructure support indicators against international best practices using a Likert scale and identify priority actions to address the double burden of malnutrition in Senegal.

**Setting:**

Senegal, West Africa.

**Participants:**

A national group of independent experts from academia, civil society, non-governmental organisations and United Nations bodies (*n* =15) and a group of government experts from various ministries (*n* =16) participated in the study.

**Results:**

Implementation of most indicators aimed at creating healthy eating environments were rated as ‘low’ compared to best practice (31 on 43, or 72 %). The Gwet AC2 inter-rater reliability was good at 0·75 (95 % CI 0·70, 0·80). In a prioritisation workshop, experts identified forty-five actions, prioritising ten as relatively most feasible and important and relatively most effective to reduce the double burden of malnutrition in Senegal (e.g. develop and implement regional school menus based on local products (expand to fourteen regions) and measure the extent of the promotion of unhealthy foods to children).

**Conclusions:**

Significant efforts remain to be made by Senegal to improve food environments. This project allowed to establish an agenda of priority actions for the government to transform food environments in Senegal to tackle the double burden of malnutrition.

Food systems in Africa are undergoing rapid transformations that are driven by a range of factors such as agricultural industrialisation, population growth, urbanisation, climate change and technological innovations^(1)^.These changes in food systems and environments have favoured, among others, a nutritional transition in many African countries marked by an increase in the prevalence of risk factors for diet-related noncommunicable diseases, with micronutrient deficiencies and hunger still persisting, thus creating a double burden of malnutrition^(1)^.

In Senegal, the double burden of malnutrition mainly affects vulnerable groups, namely women and children. The prevalence of stunting in children under 5 years of age increased from 16·0 % to 18·8 % between 2005 and 2018^(2,3)^, while the prevalence of anaemia among children under 5 years of age decreased from 83 % in 2005 to 71 % in 2017^(2,4)^. Among women aged 15–49 years, the prevalence of nutritional anaemia declined from 59 % in 2005 to 54 % in 2017, but it remains high and problematic amongst adolescent girls aged 15–19 years^(2,4)^. This reduction in nutritional anaemia was partly due to the implementation of a set of political actions during the first decade of the 21st century. These include the first Nutrition Development Policy of 2001 and the creation of the Cell against Malnutrition (CLM) in 2001. Those enabled the coordination and implementation of nutrition policies and interventions implemented at national and community levels such as the Nutrition Reinforcement Program in 2002 and the Senegal National Food Security Strategy of 2002–2015. Furthermore, health policies targeting maternal and child health with free healthcare initiatives were created in 2003 by the Minister of Health.

At the same time, the emergence of risk factors for diet-related non-communicable diseases such as high blood pressure, overweight and obesity has increased^(4,5)^. According to the results of a national survey carried out in 2015 among people aged 18–69 years, the prevalences of overweight and obesity were 22·1 % and 6·4 %, respectively, with higher rates in women^(6)^. In addition, a study carried out in school-aged children found a prevalence of overweight and obesity of 15·8 % and 4·6 %, respectively, in 2015^(7)^. Finally, nationally, 45 % of women and 27 % of men suffer from high blood pressure compared to 25 % of the population 25 years ago^(4,5)^. In terms of mortality, 80 % of deaths in Senegal are due to CVD, and these represent the second cause of death in the country after malaria, and the first amongst adults treated in health institutions in Dakar^(5)^. Finally, diabetes affected 6 % of women and 9 % of men in 2014^(8)^.

The Senegalese government has, however, shown some political commitment to prioritise improving population nutrition^(9–12)^. Indeed, a set of nutrition policies such as the National Nutrition Development Policy Document in 2015–2025, the National Support Program for Food Security and Resilience 2018–2022, and the Multisectoral Strategic Plan for Nutrition 2018–2022 were implemented to reduce undernutrition in the country. In addition, the institutionalisation of nutrition within the twelve sectors having a direct or indirect impact on nutrition and the involvement of local communities in nutrition projects and programmes are factors favourable to the practice of public health nutrition and the promotion of healthy eating in Senegal^(10–12)^. These twelve sectors work together with a view to achieving the nutrition goals of the Multisectoral Strategic Plan for Nutrition 2018–2022. While research on food environments for the prevention of obesity and diet-related noncommunicable diseases is considered a public health priority in many countries^(13)^, little efforts have been made in Senegal to improve the availability, accessibility, affordability and promotion of healthy foods in local food environments.

To support the countries of French-speaking West Africa in the fight against hunger and malnutrition in all its forms, FAO organised in Lomé in November 2016 a capacity building workshop for the various government sectors concerned, and for institutions in African countries French-speaking in order to help them develop, implement and monitor and evaluate Food-Based Dietary Guidelines (FBDG). To this end, a Senegalese delegation took part in this workshop and defined an action plan for the development and implementation of FBDG under the coordination of the Ministry of Health and Social Action (MHSA). Then, the MHSA set up a multisectoral steering committee responsible for ensuring the process of developing the FBDG. This committee brought together actors from government, academia and civil society and represented a great opportunity to reach out and raise awareness about the role of food environments amongst all actors involved in nutrition in the country, and to conduct an evaluation of all public policies and actions developed and implemented by the government aimed at creating healthy food environments in Senegal. This committee acted as facilitator for the project and was a great way to identify experts from various domains to participate in the workshop.

In this context, the main aim of this study was to assess the level of implementation of public policies and infrastructure support for policy development and implementation aimed at creating healthy food environments and to set up an agenda of priority actions to address the double burden of malnutrition in Senegal. To achieve this aim, three specific objectives are defined: (i) create and validate by national authorities a review document of all public policies and infrastructures aimed at improving food environments in Senegal; (ii) assess the level of implementation of public policies and infrastructure aimed at improving food environments against international best practices by national experts; and (iii) identify a list of priority policy actions by national experts to improve food environments and reduce the double burden of malnutrition in Senegal.

## Materials and methods

This study used the Healthy Food Environment Policy Index (Food-EPI) tool and process developed by the International Network for Food and Obesity Research, Monitoring and Action Support (INFORMAS) to assess Senegal food environment policies compared to international best practices^(13,14)^. Food-EPI is a preferred, high-quality instrument for the evaluation of public health nutrition policies. Compared to other tools, Food-EPI uses a process which strongly engages with experts and policymakers which enables greater awareness about the importance of healthy food environments^(15)^. The Food-EPI tool and process are described in detail in previous publications^(13,14,16)^.

### Food-EPI tool

**Fig. 1:**
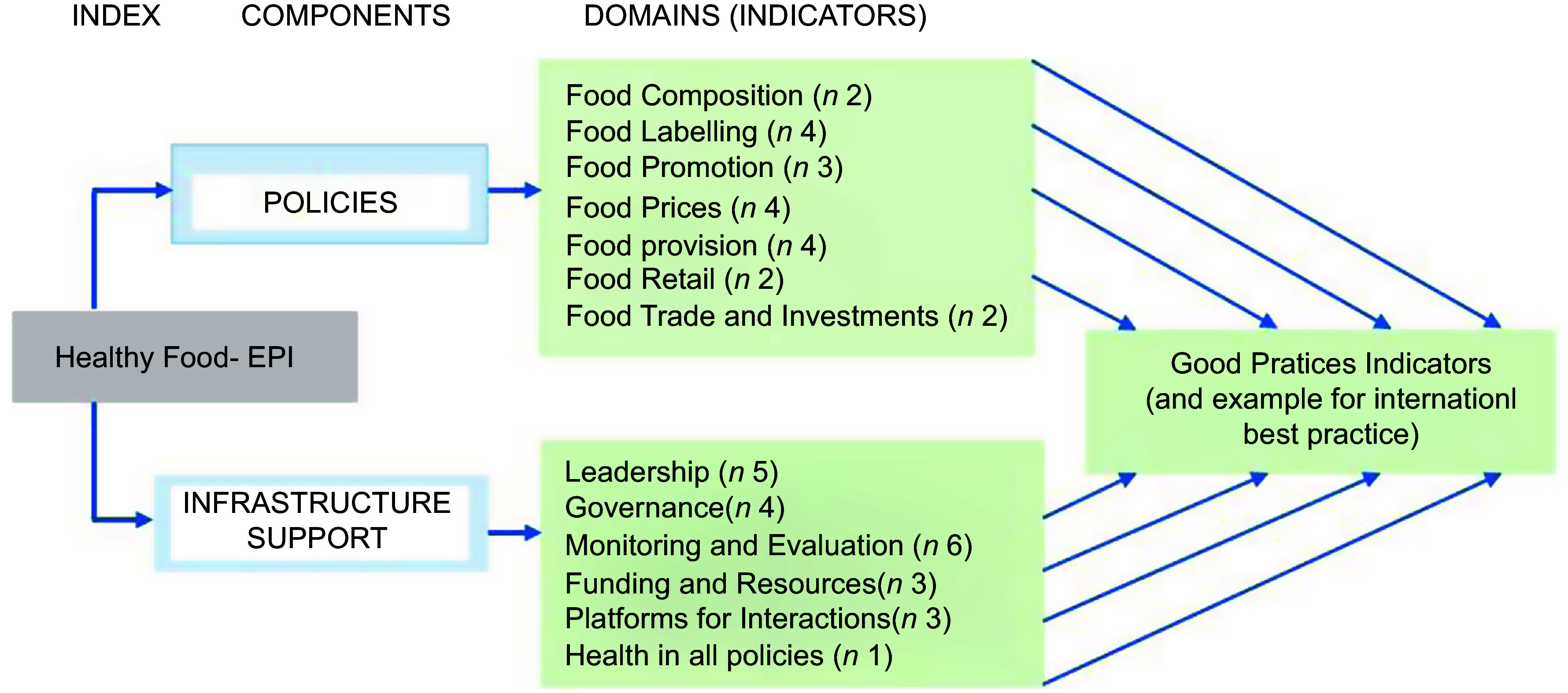
Components, domains and indicators (*n* 43) of Food-EPI tool used in Senegal

The Food-EPI assesses government actions within two primary components: (1) policies and (2) infrastructure support for development and implementation of policies. Within these components, seven policy ‘domains’ (food composition, food labelling, food promotion, food prices, food provision, food retail, and food trade and investment) and six infrastructure support ‘domains’ (leadership, governance, monitoring and intelligence, funding and resources, platforms for interaction, and health-in-all-policies) are assessed, using a set of forty-seven ‘good practice indicators’ for specific policy areas relevant to each domain (Fig. 1). However, the Food-EPI indicators were developed with a focus on obesity and diet-related noncommunicable diseases prevention, while many countries like Senegal are faced with the double burden of malnutrition. The tool thus does not include other policy areas relevant to nutrition such as GM organisms, food safety, food production, food security, undernutrition, micronutrient deficiencies, and breast-feeding or infant formula, and environmental/climate change policies.

### Food-EPI implementation process

**Fig. 2:**
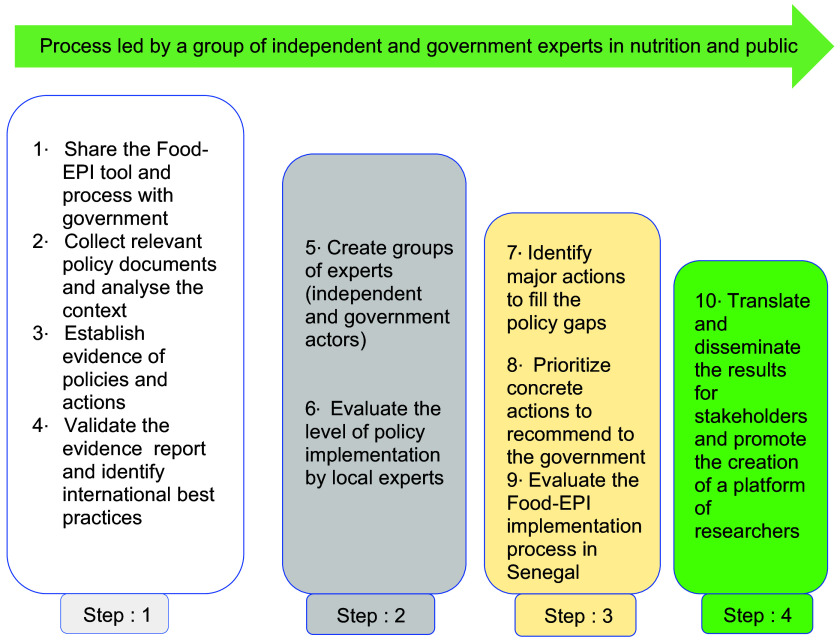
Process for assessing the level of implementation of government policies and infrastructures on food environments based on international best practices using the Healthy Food Environment Policy Index (Food-EPI)^(13)^

In accordance with the protocol developed by INFORMAS, the Food-EPI process was carried out in Senegal in nine activities grouped into four major steps (Fig. 2):

#### Step 1: Creation of an evidence-based document on food environment policies

##### Sharing the Food-EPI tool and process

The first step in the process was to share the Food-EPI tool and process with members of the multisectoral technical committee responsible for the National FBDG. This was done during a meeting organised by the MHSA which coordinates the FBDG development process. This meeting was used to present the Food-EPI research project to local authorities, including the CLM which is an inter-governmental platform that facilitates the development of nutrition policies and coordinates nutrition interventions at the national level, and to the multi-stakeholder SUN civil society platform. This step guaranteed governmental collaboration and the appropriation of results at the end of the project.

##### Policy review and analysis of the context

Collecting relevant national policy documents was the second step in the process. This collection was facilitated by the various sectors of government, particularly the CLM, which made all the important documents available. In addition, meetings with some officials from the various government sectors (agriculture, livestock, fisheries, education, economy and finance, trade and industry, etc.) were organised to collect additional information and documents. These were also facilitated by a memo issued by the MHSA to the actors concerned but also with the support of the CLM who mobilised the sectors that are involved in the development and implementation of the multisectoral nutrition strategic plan 2018–2022. These documents made it possible to describe the general context of Senegal and to collect data on policy development and implementation for a list of forty-three indicators of good practice as defined by the Food-EPI tool. Overall, we did not experience problems accessing the required documents. The only difficulty was getting access to documents that provided details on the specific cost of nutrition interventions regarding the ‘Fund1 indicator’ of the domain funding and resources. However, four indicators of good practice were not included in the report because they were deemed unsuitable for the Senegalese context by the research project team. These are Q20-Retail 3 and Q21-Retail 4, Q45-Platf 4 and Q47-Hiap2.

##### Evidence document development

The third step aimed at creating an evidence-based document on public policies and government actions according to the forty-three good practice indicators on food environments in Senegal. This phase lasted 6 months (November 2017 to April 2018). The documents were been classified into three frameworks: (i) Policy : these are political guidelines for nutrition or health or legislation (Law or Decree) in the field of nutrition; (ii) Strategic: these are documents which outline the strategic political axes or which operationalise the orientations of the policies; and (iii) Operational : these are often: (a) activity reports from different sectors sensitive or specific to nutrition, b) reports of national nutrition or health surveys and (c) nutrition programmes.

##### Evidence document validation

The evidence document was shared and validated by members of the multisectoral technical committee of FBDG including government actors. Lastly, for each of the forty-three indicators, the corresponding international best practices ‘benchmarks’ as defined by INFORMAS was identified and translated into French for evaluation purposes.

#### Step 2: Assessment of the level of policy implementation

##### Selection of experts

A panel of experts was convened from the various areas of food and nutrition research, practice, and others in Senegal by the team of researchers. A total of sixty-one experts were invited, thirty-six were from academia, civil society, non-governmental organisations and United Nations bodies (here refers to as ‘independent’) and twenty-five of which were from government (group B). Indeed, by involving government actors in the Food-EPI implementation process, the project team aimed at promoting the participatory approach and subsequent appropriation of the results. All experts completed a written informed consent form and declared their interests. Individuals with industry affiliations were purposefully excluded from the sample to avoid conflict of interest in rating policy actions.

##### Evaluation process

The evaluation workshop was organised by the MHSA in collaboration with the Nutrition Laboratory of Cheikh Anta Diop University. This workshop brought together the two groups of experts in the same room. In addition to these experts, this workshop hosted an expert from the INFORMAS network who supervised the activities. Before the assessment workshop, experts received all documents in both hard and electronical copies and were invited to review the evidence document. At the workshop, experts received a brief information session on the Food-EPI methodology and tool. For each good practice indicator, the current evidence of implementation by the Senegal government for the indicator was presented followed by the corresponding international benchmark example. Experts were then asked to take 2–3 min and rate the current level of implementation of each indicator of good practice against international best practices using a Likert scale (from 1 = less than 20 % implemented compared to best practice to 5 = 80–100 % implemented compared to best practice). The experts completed the ratings manually on a paper form. For some indicators, discussions and clarifications were necessary to harmonise understanding among experts. After the workshop, all the scoring forms were collected and evaluated by the research team.

#### Step 3: Prioritising actions

After the assessment workshop, identifying and prioritising actions was the third step of the Food-EPI process. The same experts who took part in the evaluation workshop were invited to identify and prioritise actions to recommend to the government to improve food environments. This second activity was carried out as a 2-d workshop.

The first day of the workshop was devoted to identifying concrete actions for policies (*n* 14) and infrastructure support *(n* 31) that could be recommended to the government to improve food environments. During the workshop, the research project facilitator presented a summary of the provisional results of the evaluation workshop and the methodology for identifying and prioritising actions. The principle was to identify actions to fill the gaps identified in the implementation of policies, strengthen the policy actions already implemented or choose actions that could consider the double burden malnutrition by improving food environments. Actions were identified by two mixed working groups. Each group consisted of independent experts and government experts with a moderator, a rapporteur and a time controller. Groups identified actions separately by component and an indicator could have several or no actions at all. Discussion and interaction between actors took place during this first day of the workshop. The number of actions to be identified was left to the discretion of the working groups. The workshop ended with the presentation of the identified actions of each group followed by discussion and then validation by the large group of the common list of actions to be prioritised.

The second day of the workshop was devoted to the prioritisation of actions. Each expert was asked to rate and prioritise all proposed actions for both the policy and infrastructure components using a Likert scale (1 to 5) for each of the three criteria: importance, achievability and likely effectiveness to reduce the double burden of malnutrition (Table 1). The number 1 meant lower importance, achievability and effect on the double burden of malnutrition and the number 5 meant higher importance, achievability and effect on the double burden of malnutrition. Last, during this workshop, a questionnaire (annex 1) was submitted to experts to assess their knowledge and experience after the Food-EPI tool and process.

**Table 1:** Criteria for prioritising actions to government, Food-EPI Senegal, 2019

Importance (C1)	Achievability (C2)	Potential effect of the action on the double burden of malnutrition (C3)
Need: The size of the implementation gap Impact: The effectiveness of the action on improving food environments and diets (including reach and effect size). Equity: Progressive/regressive effects on reducing food/diet-related health inequalities	Feasibility: How easy or hard the actions is to implement Acceptability: The level of support from key stakeholder including government, the public, public health and industry Affordability: The cost-effectiveness of the action	Beneficial effect: Does the implementation of the action have a beneficial effect on the double burden of malnutrition Aggravating or neutral effect: Does the action increase the risk of other forms of malnutrition or NCD or not?
Other positive effects: (e.g. on protecting rights of children and consumers) (e.g. regressive effects on household income, infringement of personal liberties)	Efficiency: The cost-effectiveness of the action	

NCD, noncommunicable diseases.

Each proposed action was ranked from: (i) higher importance to lower; (ii) high probability achievability to lower and (iii) greater potential beneficial effect at a lower or neutral on the double burden of malnutrition using the 5 to 1 scale (i.e. attribution number from 5 to 1).

#### Step 4: Dissemination of results

The results on the level of implementation of policies and priority actions were presented and shared during a workshop where national and regional experts in food and nutrition were invited from various sectors including academia, civil society, government, private sector and UN agencies. At the meeting, experts from Benin, Togo, Cote d’Ivoire and Burkina Faso were invited and led to the creation of a regional platform (REPSAO) aimed at raising awareness and stimulating research on food environments and food systems in Francophone West Africa.

### Statistical analysis

Descriptive statistics were generated using Microsoft Excel®. The mean rating for each good practice indicator was used to determine an overall percentage level of implementation at the group level. Mean ratings were then categorised into the following levels of implementation based on the following cut-off points: >75 % = ‘High’; 51 to 75 % = ‘Medium’; 26 to 50 % = ‘Low’; and ≤ 25 % = ‘Very little implementation – if any’. Differences in ratings based on experts’ background, that is, ‘government’ *v*. ‘non-government’ were tested. Inter-rater reliability agreement among these two groups of participants was assessed using the Gwet AC2 coefficient with the AgreeStat software (Agreestat 2013.1, Advanced Analytics).

**Fig. 3:**
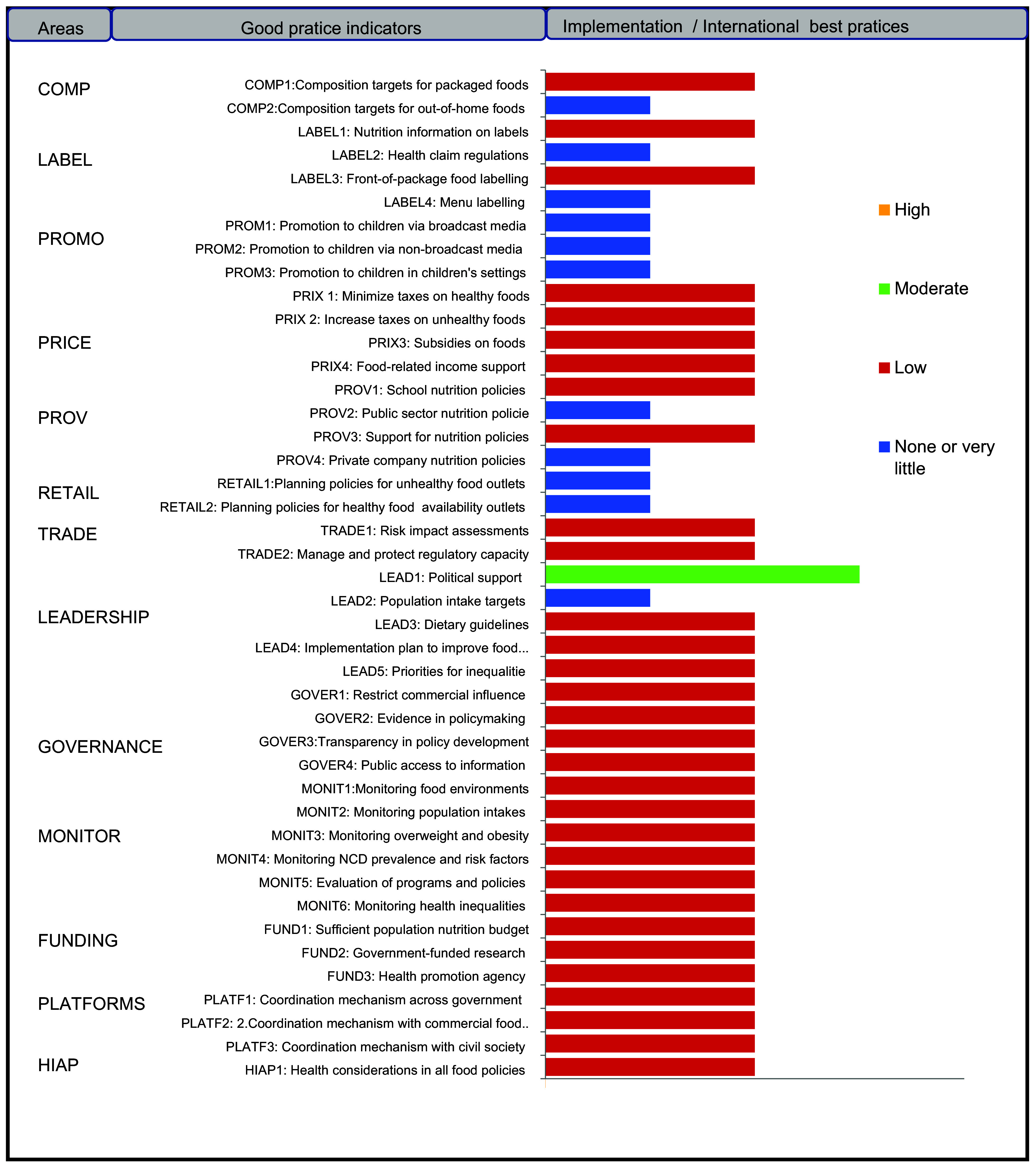
Level of implementation of policies compared to international best practices for 43 Food Environment Policy Index (Food-EPI) indicators within 7 policy and 6 infrastructure support domains

**Fig. 4:**
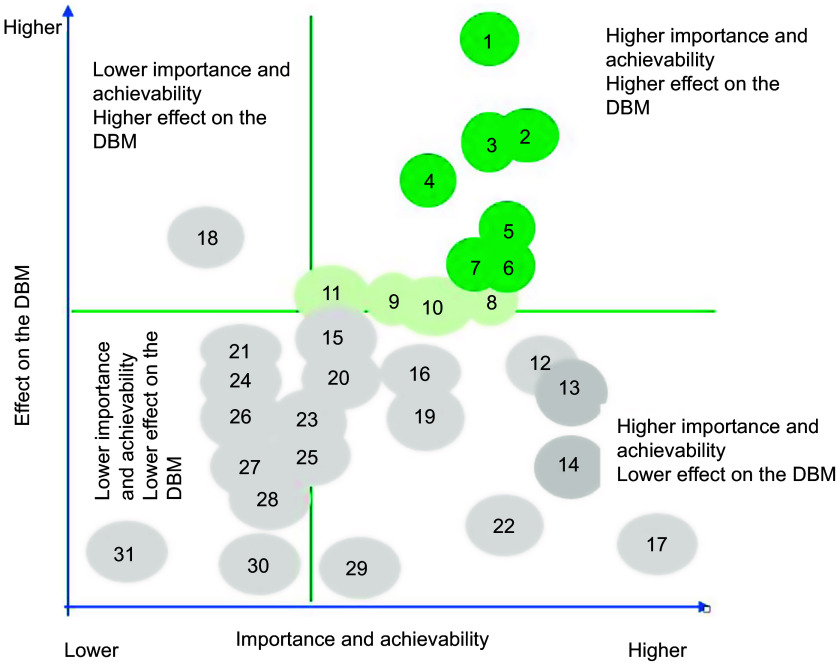
Importance, achievability and effect on the double burden of malnutrition of policy actions for the Senegalese government according to expert scores, and the Food-EPI domains which the policy falls within, Food-EPI Senegal, 2019. Detailed descriptions of proposed policy actions can be found in Table 3. DBM, double burden of malnutrition

For the prioritisation of proposed actions, weights allocated to importance, achievability and effect on the double burden of malnutrition were applied to individual scores, and mean scores for importance and achievability were then summed for each proposed action to determine one only criterion. Then, the same thing was applied for the potential effect on reducing the double burden of malnutrition. Actions were then ranked from lower to higher priority (Tables 2 and 3). Average points on importance and achievability and effect on the double burden of malnutrition scales were mapped using a four-quadrant scatter graph (Figs. 3 and 4). The actions were divided into four groups: (i) ‘relatively higher importance and achievability and relatively higher effect on the double burden malnutrition’ group; (ii) ‘relatively higher importance and achievability and relatively lower effect on the double burden malnutrition’ group; (iii) ‘relatively lower importance and achievability and relatively higher effect on the double burden malnutrition’ group; and (iv) ‘relatively lower importance and achievability and relatively lower effect on the double burden malnutrition’ group (Figs. 3 and 4). The higher the points allocated to these two criterions, the more likely the proposed policy actions were assigned at the upper-right quadrant of the scatter graph.

**Table 2:** Policy documents identified and included for review, Food-EPI Senegal, 2019

Policy framework		
*n*	Title of document	Date published
1	Agriculture Sector Policy Letter 2018–2022	2018
2	Livestock Development Policy Letter 2017–2021	2017
3	National Quality Policy of the Senegalese Ministry of Industry and Mines	2017
4	Fisheries and Aquaculture Sector Development Policy Letter 2016–2023	2016
5	Policy letter from the Environment and Sustainable Development Sector 2016–2020	2016
6	Sector Policy Letter of the Ministry of Trade	
7	National Nutrition Development Policy Document 2015–2025	2015
8	Emerging Senegal Plan	2014
9	National policy for infant and young child feeding	2014
10	DECREE No. 2005-913 of 12 October 2005 making the application of the Codex standard on the labelling of prepackaged foodstuffs compulsory	2005
11	Customs clearance of Alcohols, Carbonated Drinks, Alcoholic Drinks and Alcoholic or Alcoholic liquids.	
12	Law No. 83-20 of 28 January 1983 relating to advertising	1983
13	Decree No. 68-507 of 7 May 1968 regulating the control of products intended for human and animal food of the Ministry of Trade	1968
14	Decree No. 68-508 of 7 May 1968 setting the conditions for research and recording of breaches of Law No. 66-48 of May 27, 1966 of the Ministry of Trade	1968
15	Law No. 66-48 of 27 May 1966 relating to the control of food products and the repression of fraud by the Ministry of Trade	1966
Strategic framework		
16	Multisectoral Strategic Plan for Nutrition (MSPN) 2018–2022	2018
17	Senegal National Food Safety Strategy 2018–2035	2018
18	Strategic Plan 2017–2021 for the fortification of foods with micronutrients of the Senegalese Committee for the Fortification of Foods with Micronutrients	2017
19	National Food Safety Emergency Response Plan in Senegal	2017
20	National Social Protection Strategy 2016–2035	2016
21	National Strategy for Equity and Gender Equality 2016–2026	2016
22	Food and Nutrition Strategic Plan 2016–2020 of the Ministry of Health	2016
23	National Food Security and Resilience Strategy 2015–2035	2015
24	National Strategic Plan for Community Health 2014–2018 of the Ministry of Health	2014
25	Strategic plan for the development of Universal Health Coverage in Senegal 2013–2017	2013
26	National Strategic economic and Social Development 2013–2017	2013
27	National Health Development Plan 2009–2018	2009
Operational framework		
28	National Support Program for Food Security and Resilience 2018–2022	2018
29	Sectoral Action Plans for sectors implementing the MSPN 2018–2022	2018
30	Report of the school canteens of the Ministry of National Education	2018
31	Demographic and Health Surveys 2005–2018	2018
32	Decentralised Evaluation. PAA Africa programme in Senegal’s Kedougou region. September 2013–July 2016; 2017 PAM/FAO	2017
33	Report of the Joint Agricultural Sector Review	2017
34	Report of the National Food Security Survey in Senegal	2016
35	Report of the Joint Agricultural Sector Review	2016
36	Guide to school canteens 2016 version from the Ministry of National Education	2016
37	Activity report on the Ministry of Health’s Tumor Registry	2016
38	Traceability and impact of agricultural subsidies	2015
39	Senegalese Agriculture Cadence Acceleration Program 2014–2017	2014
40	Global Vulnerability, Food Security and Nutrition Analysis 2014	2014
41	Assessment of food security and agricultural markets in Senegal	2014
42	National Family Security Scholarship Program	2013
43	Assessment of the impact of school feeding programmes on the internal efficiency of schools, cognitive acquisitions and the learning capacities of students in rural primary schools in Senegal.	2013
44	United Nations System Standing Committee on Nutrition (UNSCN). National Policy Analysis	2013
45	Receipt book for school canteens of the Ministry of National Education	2012
46	Guide for the establishment and management of school canteens of 2011 from the Ministry of National Education	2011
47	Senegal health system assessment report 2009	2009

**Table 3:** Priority policy actions recommended to the government by local experts based on their importance, achievability and effect on the double burden of malnutrition, Food-EPI, Senegal, 2019

1	PROV1a	Develop and implement regional school menus based on local products (expand to fourteen regions)
2	COMP2a	Define a model of good practices and nutritional standards by focusing on the content of nutrients of concern in foods offered (e.g. fried foods) in fast-food establishments.
3	PROV1b	Implement nutritional standards for school meals
4	LABEL1a	Introduce obligatory legislation for nutrients (energy, saturated fat, total fat, protein, carbohydrates, sugars and salt) on packaging
5	PROM3	Put in place regulations that restrict the promotion of unhealthy foods in the living environment of children.
6	PROM1a	Use the media (public and private) to convey messages for healthy eating during peak hours
7	PROM1b	Establish regulations on the promotion of unhealthy foods through the audiovisual and broadcast media
8	COMP2b	Strengthen the control system of fast-food establishments to ensure compliance with nutritional standards for nutrients of concern in foods
9	PRIX4	Supplement the National Family Grant Program with nutrition and health education interventions
10	PROV4	Set up a support, training and follow-up system by promoting the supply and offer of healthy foods and meals in private food service establishments (school canteens, etc.).
11	RETAIL1	Develop laws to regulate the establishment of fast-food establishments
12	LABEL1–2	Transcribe codex nutrient standards and claims into national legislation
13	LABEL1b	Strengthen controls to respect the labelling and composition of food
14	COMP1	Increase the range of standards applied to processed products, particularly those most consumed by the population, and make their application compulsory
15	COMP2c	Target restaurant owners to encourage them to respect this model through labelling
16	PRIX1	Reduce taxes on local fruits and vegetables
17	COMP1	Updates the food composition table
18	RETAIL1	Regulate the distance between schools and fast-food outlets
19	LABEL2	Strengthen the body responsible for verifying claims on food products (the manufacturer must himself provide proof of his claims)
20	PRIX3	Orient subsidies towards healthy foods
21	RETAIL2	Develop mechanisms to reduce the exposure of children to unhealthy foods and promote the accessibility of healthy foods in different types of establishments
22	PROV2	Establish nutritional standards for meals offered in universities, hospitals and prisons
23	PROV3	Set up support and training systems for public structures to promote the supply of healthy foods
24	PRIX2	Establish mechanisms to reduce the affordability of unhealthy foods
25	PRIX1	Conduct an analysis of the fiscal space available for the promotion of healthy foods correlated with a study on the economic impact of obesity and chronic disease
26	PROV3–4	Strengthen the capacity of restaurateurs in the public and private sectors on food choices and their contribution in relation to nutritional status
27	PROV4	Develop a label for restaurateurs and suppliers of healthy and adequate food. This label will be required when they supply food to schools and other public sectors
28	LABEL3	Encourage the use of colour codes to better inform consumers about the quality of food
29	TRADE2	Strengthen existing trade regulations and the control of their application
30	TRADE1	Conduct impact studies of trade agreements on the food environment of populations
31	PRIX4	Directing income support programmes for poor households towards the provision of healthy foods

## Results

### Policy evidence document

A total of forty-seven governmental documents were collected, analysed and classified for this study (31·9 % are from the policy framework (*n* 15), 25·5 % are form the strategic framework (*n* 12) and 42·5 % are from operational framework (*n* 17) (Table 4). These documents were used to create an evidence report validated by government authorities and members of the multisectoral committee for National FBDG and served as a reference document for the evaluation and prioritisation workshops.

**Table 4:** Priority infrastructure support actions recommended to the government by local experts based on their importance, achievability and effect on the double burden of malnutrition, Food-EPI, Senegal, 2019


1	PROM1	Measure the extent of the promotion of unhealthy foods to children
2	COMP1–2	Conduct a national survey to assess the food consumption of the population
3	LEAD3	Complete the development of National Food-Based Dietary Guidelines
4	MONIT2a	Include children 6–9 years old in the periodic monitoring of nutritional status
5	PLATF1a	Strengthen the monitoring of non-communicable diseases of the national strategic plan to control NCD by integrating multisector
6	LEAD4a	Develop an implementation plan for National Food-Based Dietary Guidelines
7	MONIT2b	Establish a mechanism for periodic monitoring by surveys of food intake every 10 years
8	LEAD4	Introduce policies on healthy food environments (recommended by the Food-EPI tool) into the existing nutrition strategy
9	MONIT1	Take stock of existing monitoring and evaluation systems (e.g. school canteens) in order to strengthen the system
10	PLATF3	Strengthen the framework for consultation between the state and civil society (NGO, consumer associations, etc.) in order to better take charge of food policies and other interventions to improve the nutrition of populations.
11	PLATF1b	Set up the two SUN platforms for academics and the private sector
12	GOVER2	Disseminate food policies and research documentation to the population in a simple way
13	FUND1	Use the results of an analysis of the budget space available for the promotion of healthy foods correlated with a study on the economic impact of obesity and chronic disease to determine the budget needed for health promotion
14	GOVER1	Develop mechanisms to manage potential conflicts of interest in the development of food policies

NCD, noncommunicable diseases.

### Characteristics of experts

Sixty-one experts in public health and nutrition were invited, thirty-one took part in the evaluation workshop for a participation rate of 50 %. The experts were classified into two groups. Group A was made up of independent experts (academics, civil society and United Nations) (*n* 15) and group B was made up of government experts (*n* 16). Among the thirty-one experts, 45·2 % were women (*n* 14) and 54·8 % were men (*n* 17). Experts came from a variety fields of research and practice, including dietetics, nutrition, public health, health policy, health economics, trade, agriculture and food security, among others.

### Level of policy and infrastructure support implementation

**Fig. 5:**
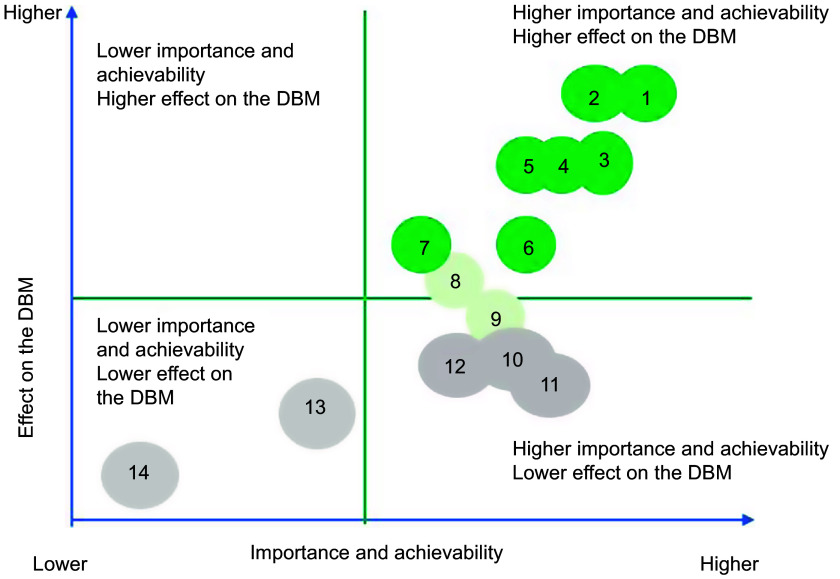
Importance, achievability and effect on the double burden of malnutrition of infrastructure support actions for the Senegalese government according to expert scores, and the Food-EPI domains which the policy falls, Food-EPI Senegal, 2019. Details descriptions of proposed infrastructure actions can be found in Table 4. DBM, double burden of malnutrition

Implementation of most indicators aimed at creating healthy eating environments were rated as ‘low’ (31 on 43, or 72 %) by local experts. In the policy domain of food promotion and food retail, all indicators were assessed at very low if any implementation compared to international best practices (Fig. 5). In the infrastructure support component, one indicator relating to strong and visible government support for public nutrition in the area of leadership (LEAD1) was assessed with a ‘medium’ level of implementation. Finally, none of the indicators were assessed with a high level of implementation compared to best practices. The overall inter-rater reliability was 0·75 (95 % CI 0·70, 0·80). Inter-rater reliability was also calculated separately for both groups: (group A) independent experts 0·77 (95 % CI: 0·71, 0·84) and (group B) government experts 0·64 (95 % CI 0·64, 0·80). Indeed, 14 % of the indicators (six out of foty-three) were rated very differently between the two groups; government experts tended to give some indicators a higher score. Four indicators were rated medium in group B, compared to one indicator in group A (Table 5). In the prioritisation workshop, experts identified and prioritised forty-five priority actions (thirt-one actions policy and fouteen infrastructure support actions), including ten main actions to improve food environments in Senegal and reduce the double burden of malnutrition (Tables 2 and 3). These ten main actions recommended to the Government of Senegal are those of highest importance and achievability and most significant potential to reduce the double burden of malnutrition (Figs. 3 and 4). Finally, these ten main actions were for indicators rated as ‘very little’ (4 on 10, or 40 %) or ‘low’ (6 on 10, or 60 %). Since there are no existing indicators specifically related to undernutrition in the Food-EPI tool, the issue of double burden was only considered in the identification and prioritisation of actions, not in the indicators to themselves (Tables 2 and 3).

**Table 5:** Indicators evaluated differently in the two groups among the forty-three indicators, Food-EPI Senegal, 2019

Indicators	Independents (*n* 15)	Government (*n* 16)
LABEL1: Nutrition informations on labels	Low	Medium
PROM3: Promotion to children in children’s settings	Very low	Low
RETAIL2: Planning policies for healthy food availability outlets	Low	Very low
LEAD2: Population intake targets	Very low	Low
PLATF3: Coordination mechanism with civil society	Low	Medium
HIAP2:Health considerations in all policies	Low	Medium

### Capacity building

Twenty-five questionnaires were completed by experts including thirteen in the group of independent experts and twelve in the group of government. Overall, 96 % of participants agreed or strongly agreed that they had increased their knowledge of food environments and related food and nutrition policy after the Food-EPI process. A majority (92 %) had increased their knowledge of current best practice and international government actions as well after this experience. Overall, 84 % agreed or strongly agreed that the project was likely to have a policy impact in their country and 92 % agreed that it was important to repeat the same study over time to assess progress. Finally, 76 % said they would be willing to participate in the assessment process again 2 or 3 years later.

## Discussion

In our study, thirt-one participants among the sixty-one invited expert evaluators participated in the evaluation workshop, for participation rate of 50 %. This rate is comparable and even higher than that of other countries. Indeed, the Food-EPI evaluations in Australia, Canada and Thailand, respectively, had a participation rate of 70 %, 64 % and 59 %. On the other hand, Chile, Ghana, Singapore, Kenya, Mexico and South Africa recorded participation rates varying, respectively, from 28 % to 46 %^(17–19)^.

The present study revealed that the groups of independent and governmental experts made different assessment of the level of policy implementation and infrastructure support aimed at creating healthy food environments in Senegal. Indeed, the variations were probably due to inherent differences between the groups of evaluators including their role^(20)^ or their potential bias associated with their position of responsibility^(21,22)^. Indeed, the inter-rater reliability of the group of independent experts was higher (0·77) than that of government experts (0·64). In addition, government experts assigned a higher percentage of implementation to several indicators compared to independent experts. Our results are like studies in Thailand, Mexico and New Zealand that involved government actors in the assessment process^(17,23)^. In our study, experts from different sectors of government were targeted to participate in the assessment workshop and were sometimes represented by representatives meaning this group of experts was probably not as homogeneous as the group of independent experts.

In our study, 48 % (ten out of twenty-one) of the good practice indicators of the ‘policy’ component recorded a ‘very low or even non-existent’ level of implementation (i.e. ≤ 25 %) against 5 % (one out of twenty-two) in the ‘infrastructure support’ component compared to international best practices (Fig. 5). Elsewhere, as in Guatemala, 83 % of the indicators of good practice in the ‘policy’ component recorded a ‘very low or even non-existent’ level of implementation^(17)^. On the other hand, 12·5 % (two out of sixteen) of the good practice indicators were rated ‘very low or even non-existent’ in Kenya, while in Ghana no good practice indicator of the ‘policy’ component was rated ‘very low or even non-existent’^(18,19)^. The country with the best evaluation is that of Chile, whose implementation of several indicators of good practice of the ‘policy’ component were evaluated with a ‘high’ score^(17)^.

In the ‘infrastructure support’ component, 90% (twenty out of twenty-two) of the good practice indicators were assessed with ‘low’ implementation (i.e. between 26% and 50%). This percentage was 86% (nineteen out of twenty-two) in Kenya and 65% (thirteen out of twenty) in Ghana^(18,19)^. In these two countries, the number of good practice indicators with a ‘medium’ level of implementation was, respectively, 30% (six out of twenty) in Ghana, 13·6% in Kenya (three out of twenty-two) against 4·5 % (one in twenty-two) in Senegal^(18,19)^. However, in Singapore and New Zealand, 29% and 21%, respectively, of the infrastructure support indicators were rated ‘high’ compared to international best practices^(17)^.

Overall, none of the forty-three indicators assessed from the two components of our study recorded a ‘high level of implementation’ (i.e. above 75 %) which means that there is still work to be done to strengthen public policies relating to the creation of healthy food environments in Senegal. These results are also consistent with several studies that have assessed different areas of the food environment in many low- and middle-income countries through the use of Food-EPI^(17,24–26)^. This indicates that, globally, few low- and middle-income countries countries have implemented comprehensive policies and infrastructure to foster healthier food environments and support healthier food choices^(17)^. However, Ghana and Kenya, respectively, recorded more indicators of good practice with a ‘medium’ level of 22 % (eight out of thirty-six) and 10·5 % (four out of thirty-eight) compared to Senegal, where only one indicator (political support for nutrition) has been graded with a ‘medium’ level of implementation^(18,19)^ but shows a promising future for the country.

Nevertheless, the political context of Senegal is very favourable to the promotion of healthy food environments given the recent commitment of the public authorities (PNDN, 2015, and PSMN, 2018) and the active participation of government experts during the implementation process implementation of this study.

Our study introduced a third criterion in the process of prioritisation of actions by identifying actions having a potential effect on the double burden of malnutrition. Such an approach was an innovation in the implementation of Food-EPI in the low- and middle-income countries where the double burden of malnutrition poses a real public health problem. During the prioritisation workshop, experts prioritised eight major political actions among the thirty-one actions of the ‘policy’ component and six major priority actions among the fourteen priority actions of the ‘support for infrastructure’ component as having a potential direct or indirect effect on the double burden of malnutrition. These include (i) developing regional school menus based on local products at the level of the fourteen regions; (ii) setting up nutritional standards for school meals; (iii) regulating the promotion of food unhealthy in the living environment of children (Table 2); (iv) carrying out a study to measure the extent of the promotion of unhealthy foods for children; and (v) carrying out a national survey on food consumption within the population (Table 3). All these priority actions are considered by the WHO as having a double action on the double burden of malnutrition^(27)^.

Indeed, more than forty jurisdictions in over twenty countries have implemented sugary drink taxes, and at least eight countries have mandatory restrictions on the advertising of unhealthy foods to children via broadcast or non-broadcast media^(28)^. This demonstrates the policy momentum that is gaining in some of these policy areas that were once considered radical^(29,30)^. Although Senegal does not have national data on the consumption of sugary drinks, these regulatory policies are increasingly implemented in countries worldwide. Indeed, Senegal commenced, in 2020, a study to measure the exposure of children to unhealthy foods marketing. Reassessment of Senegalese food environment policies in 5 years (i.e. 2024) will demonstrate whether the government has successfully implemented these strategies.

The strength of the present study was the introduction of a third criterion in the prioritisation process to consider the double burden of malnutrition. Indeed, policies that were prioritised were identified as those with the potential to fill the greatest gaps in the current environment and have the greatest impact on the double burden of malnutrition, while still having high acceptability and feasibility. This has been an important innovation in the implementation of Food-EPI in Senegal unlike other studies which only used two prioritisation criteria^(17)^. The inclusion of government actors and their involvement in the Food-EPI implementation process in Senegal to promote ownership of the results was also a strong point of our study. Finally, our study brought together several multidisciplinary and multisectoral actors to assess policies and establish priority actions to improve food environments and integrated the results into the situational analysis of the development process of FBDG. This innovative approach for Senegal has made it possible to combine, in the development of FBDG, an assessment of public policies in relation to international best practices in healthy food environments.

There are several indicators that the Food-EPI study process led to significant progress in Senegal. First, the institutional ownership of Food-EPI is apparent as several good practice indicators are now included in the monitoring and evaluation system of the Multisectoral Strategic Plan for Nutrition which implements the National Nutrition Development Policy 2015–2025^(11,12)^. This nutrition policy is coordinated by the CLM, which in 2020 became the National Council for the Development of Nutrition (NCDN). The NCDN has the role of assisting the government in the development of nutrition policies and of coordinating the implementation of nutrition programmes at the national level. To facilitate this integration, Senegal has initiated a process of developing a programme called ‘Resilient food systems towards healthy diets for people vulnerable to malnutrition in Senegal’. This programme involves the National Nutrition Development Council, FAO, Solidarity Union Cooperation and other technical and financial partners. One of the strategic components of this programme relates is ‘Integrating good practices in healthy food environments and the orientations of national dietary recommendations into institutional and sectoral nutrition and food security strategies and policies’.

Second, Food-EPI results were used as a situation analysis for the preparation of FBDG which is coordinated by the MHSA. Third, some priority actions are already or currently being implemented including: (i) the creation of a national survey on food consumption carried out jointly by the Consortium for Economic and Social Research, the Nutrition Laboratory of Cheikh Anta Diop University in Dakar, FAO and the MHSA; and (ii) a research project to document the exposure to unhealthy food marketing in Senegal ensured by the Nutrition Laboratory of Cheikh Anta Diop University Dakar in collaboration with the MHSA and Council for the Development of Nutrition and the University of Montreal with funding from IDRC (2020–2023). Third, a regional platform was created to share the results of Food-EPI with researchers from African countries (Benin, Cote d’Ivoire, Togo, Burkina-Faso, Ghana and Kenya) and University of Montreal, and to develop future research on public policies and food environments in West Africa. Currently, this platform called *Research network on Public Policies and Food Systems in West Africa* is composed of five countries and is leading a new research project to conduct Food-EPI evaluations in Benin, Côte d’Ivoire, Togo and Burkina-Faso funded by the IDRC.

The main limitations of our study are related to the size of the sample, although it compared favourable to similar studies in other countries. In addition, participants were identified based on their skills and some government actors were replaced by others during the process making the group less homogeneous compared to the group of independent actors. Finally, some participants did not fully respond to the questionnaire and the length of the evaluation questionnaire was seen as a time constraint. Food-EPI was developed primarily for obesity and chronic diseases prevention, and this was considered a limitation for the participants. Future-specific indicators should be developed to specifically address the double burden of malnutrition. In this study, while the assessment itself did not consider undernutrition and focused on using the original tool, the double burden was considered when actions were identified and prioritised.

## Conclusion

This study enabled Senegal to conduct a first evaluation of the level of implementation of its public policies and government actions in relation to international best practices to create healthy food environments and has established an agenda of priority actions supported by a group of national experts aiming to improve food environments in Senegal. It also brought together and sensitised national actors around crucial public health nutrition issues and provided important contextual information for research actions. Finally, our project represents an opportunity to inform the future development of policies that can align with the FBDG.
